# A Comparison of Puncture and Continuous Pump Analgesia With Two Different Approaches to Thoracic Paravertebral Block for Thoracic Surgery

**DOI:** 10.3389/fsurg.2021.711205

**Published:** 2022-02-18

**Authors:** Qiao-wen Huang, Zhi-wei Lu, Jia-bin Li, Wen-qing Zhang, Li-wei Jiang, Zhi-jian Lin

**Affiliations:** Department of Anesthesiology, Zhangzhou Affiliated Hospital of Fujian Medical University, Zhangzhou, China

**Keywords:** ultrasound-guided, continuous thoracic paravertebral block, block plane, postoperative analgesia, thoracic paravertebral block

## Abstract

**Background:**

This study aims to compare the success rate of thoracic paravertebral block (TPVB) and the effect of postoperative analgesia between two approaches.

**Methods:**

A total of 34 patients with American Society of Anesthesiology (ASA) physical status score II–III, undergoing an optional thoracoscopic surgery, were randomly assigned to a parasagittal approach group (group P, *n* = 17) and a transverse intercostal approach group (group T, *n* = 17). The catheterization time, success rate of the puncture and catheterization, block plane and effect at the surgical site were compared between two groups. The mean arterial pressure and heart rate were recorded, as well as the cold tactile block plane and numeric rating scale (NRS) at 0.5, 2, 4, 8, 12, 24, and 48 h after surgery. The study was registered at http://www.chictr.org.cn/showproj.aspx?proj=9624 (Registration number: ChiCTR2100054642).

**Results:**

The catheterization time in group P was significantly longer than that in group T (*P* < 0.05). The success rate of catheterization in group P was lower than that in group T, but no statistical significance (*P* = 0.085). There was no significant difference in the success rate of Puncture and blocking effect of the surgical site at 30 min post-injection between two groups (*P* > 0.05). There was no significant difference in the cold tactile block plane and NRS scores during coughing between two groups at 0.5, 2, 4, 8, 12, 24, and 48 h postoperatively (*P* > 0.05).

**Conclusion:**

This study suggests that there is no significant difference in postoperative block level or pain score during coughing for thoracoscopic surgery between ultrasound-guided parasagittal and transverse intercostal approach, but the parasagittal approach takes longer and has a higher failure rate.

## Introduction

As minimally invasive surgery has developed in recent years, thoracotomy has gradually been replaced by thoracoscopic surgery. The level of pain experienced after thoracoscopic surgery is lower than that after traditional thoracotomy. In addition, the thoracic epidural block has always been the gold standard for postoperative analgesia in thoracic surgery. With the development of ultrasound technology, many studies have proposed the use of ultrasound-guided continuous thoracic paravertebral block (TPVB) to replace thoracic continuous epidural block for postoperative analgesia after thoracoscopic surgery (1, 2). Ultrasound TPVB can not only display the position of puncture needle in the whole process, accurately display the paravertebral space, ensure the anesthetic effect, but also clearly display the position of pleura to avoid perforation of pleura, so it can improve the success rate of catheterization, and may in time completely replace the thoracic epidural block. However, the difficulty of ultrasound-guided continuous TPVB lies in the thoracic paravertebral space catheterization. At present, clinical catheterization mainly uses the parasagittal approach or the transverse intercostal approach, depending on the different ultrasonic sections used at the time of puncture. However, there are few comparative studies into the difficulty and postoperative analgesic effect of these two blocking approaches for catheterization. By measuring the catheterization time, the success rate of puncture and catheterization, and the block level and postoperative analgesia, this study aims to compare the degree of difficulty and anesthetic effect of the two catheterization approaches by ultrasound in clinical practice, and explore which approach is more ideal.

## Materials and Methods

### General Information

**Figure 1:**
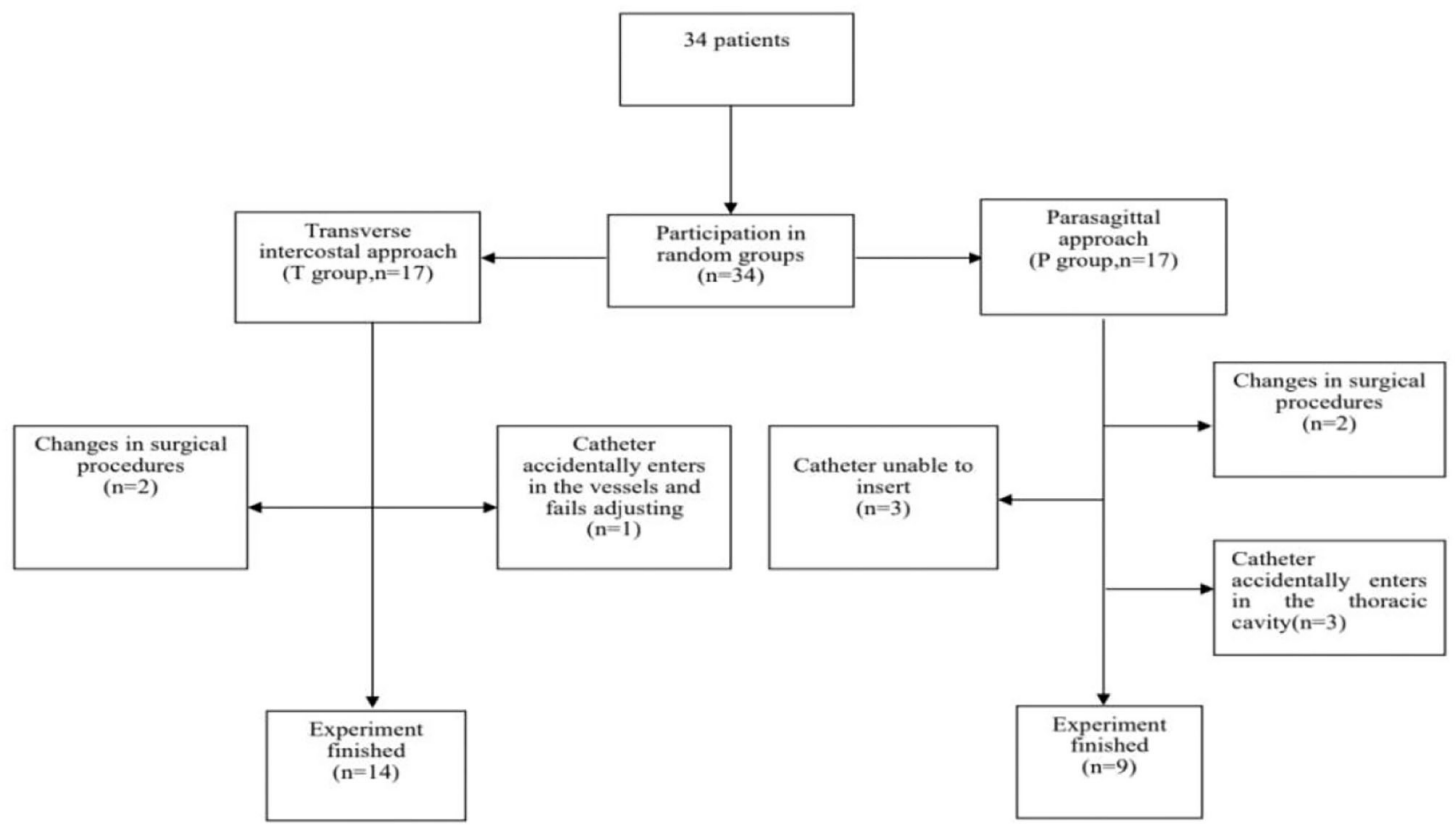
Recruitment process of subjects.

This study was conducted with approval from the Ethics Committee of Zhangzhou Affiliated Hospital of Fujian Medical University (2021LWB053) and was registered at http://www.chictr.org.cn/showproj.aspx?proj=9624 (Registration number: ChiCTR2100054642). This study was conducted in accordance with the declaration of Helsinki. All the patients gave written informed consent and were divided into two group using concealed random allocation from a computer-generated random numbers table. The inclusion criteria were as follows: patients undergoing thoracoscopic radical resection of unilateral lung cancer in our hospital and patients aged between 18 and 80 in American Society of Anesthesiology (ASA) II–III. The exclusion criteria were as follows: patients who were complicated with severe cardiopulmonary disease, abnormal coagulation function, or preoperative use of analgesic drugs. The criteria for removal from the study were as follows: patients who had total spinal anesthesia or local anesthetic toxicity or, intraoperative changes of the surgical method (e.g., single-hole thoracoscopy or conversion to thoracotomy), serious intraoperative complications, or emergency follow-up surgery. Finally, the patients were divided into a parasagittal approach group (group P, *n* = 17) and a transverse intercostal approach group (group T, *n* = 17) (Figure 1).

### Anesthetic Technique

After entering the operating theater, the patient received oxygen inhalation. Meanwhile, the blood pressure, heart rate (HR), oxygen saturation, and continuous intravenous infusion were monitored. In addition, the blood pressure was measured three times before TPVB puncture, including systolic blood pressure (SBP) and diastolic blood pressure (DBP). Mean arterial pressure (MAP) was calculated as SBP/3 + 2DBP/3. According to the formula, the baseline of MAP was calculated using average of three measurement of blood pressure before TPVB puncture.

The ultrasound-guided TPVB was performed by the same anesthesiologist (LZW) in both groups. The TPVB block method was set up as follows: both groups of patients were placed in the lateral decubitus position; an ultrasonic device (S-Nerve; FUJIFILM SonoSite Inc.) with a 6–13 MHz linear transducer was selected; and after routine disinfection and the placing of surgical drapes, the ultrasonic probe (5~13 HZ, Sonosite EdgeII, USA) was wrapped in a sterile cover. For the parasagittal approach, the ultrasound probe was placed above the fifth and sixth thoracic vertebra (T_5~6_) spinous processes with a side opening of 5–6 cm, parallel to the spine, was gradually moved toward the spine to 2–3 cm beside the spine, and then we can see flat and deep transverse processes, and then the adjacent transverse process presented a wall sign. For the transverse intercostal approach, the probe scanning method is the same as the parasagittal approach, when the ultrasound scan to the transverse process, rotate the ultrasound probe to be parallel and a little oblique to the ribs, and then we can see “a landscape sign.” An ultrasound image clearly showed the T5 transverse process, thoracic paravertebral space, and pleura in both groups.

After local anesthesia was injected at the puncture point, a 16-gauge epidural puncture needle (Disposable Epidural-Spinal combined Anesthesia Kit; Fornia Inc., Zhuhai, China) was inserted and advanced using the in-plane approach toward the TPVS. In group P, the needle was inserted from the head probe, while in group T, it was inserted from the lateral spinal probe. After verifying that needle tip arrived at the thoracic paravertebral space using ultrasound in accordance with the group allocation, the initial single shot block injection was performed by 0.33% ropivacaine 0.3 ml/kg and movement of the pleura was verified by ultrasound. Subsequently, an epidural catheter was indwelling after injection along the epidural needle, ~2 cm in depth. The needle was then withdrawn without any blood, air, or cerebrospinal fluid. The pleura was visibly down. The catheter was fixed after being properly sutured. About 20 min after performing TPVB, the block level was tested using the cold tactile method. After the level of anesthesia was checked, the routine induction of anesthesia using 2 mg/kg propofol, 0.2 mg/kg cisatracurium besilate, 0.5 μg/kg sufentanil. The insertion of double lumen tube were intubated. Total intravenous anesthesia was given to both groups of patients and one-lung ventilation been carried before surgery. During the surgery, the dosage of propofol and remifentanil was adjusted to maintain the Narcotrend value between 40 and 60. The muscle relaxant of cisatracurium was given intermittently. If the MAP and HR were higher or lower than 20% at the time of entering the operating room, vasoactive drugs were used as appropriate. All patients have chest drainage at the end of the surgery and the postoperative analgesia was turned on immediately after the operation. Both groups were treated with TPVB patient-controlled analgesia (PCA). Ropivacaine was prepared to 300 ml and 0.2%, using an Electronic Analgesic Pump (DDB-I, Nantong, Jiangsu, China), with a loading dose of 0.25 ml/kg and a maintenance dose of 0.125 ml/kg/h, a PCA of 0.125 ml/kg, and a lock time of 30 min. If the numerical rating score (NRS) score was higher than 4, flurbiprofen and/or tramadol as rescue measure were administered. On day two, 200 ml of ropivacaine, at the same concentration, was added to the analgesic pump. This study is a partial blind study, in that an individual who was unaware of the grouping was responsible for determining the effects and carrying out postoperative follow-up and data collection.

### Data Collection

The puncture and catheterization time of TPVB and cases of successful puncture and catheterization were recorded in the two groups, and the block effect of the surgical site was determined by pin pricking after TPVB. The following descriptors were used: excellent, completely painless when pin pricking; good, a decrease in pin pricking pain or only slight pain felt; and poor, unable to bear pin pricking pain. (An “excellent” needle-punching effect and a “good” judgment were regarded as a single successful TPVB puncture.) Unobstructed catheterization (2 cm) was conducted by pin pricking after the TPVB single block. Successful catheterization was also confirmed when the catheter did not penetrate the pleura during thoracoscopy. If the catheterization time exceeded 30 min, it was considered to be unsuccessful and brought to an end. MAP and HR were recorded at the time of entering the operating room, surgical skin incision, and 30 min after surgery. The cold tactile block plane was measured in the two groups at 30 min after injection and 0.5, 2, 4, 8, 12, 24, and 48 h after surgery, and the pain scores while resting and coughing were measured at the same time intervals using the NRS; the dosage of flurbiprofen and tramadol during 24 and 48 h after surgery; complication of TPVB, such as pleural puncture, local anesthetic toxity, or intrathecal injection.

### Data Analysis and Statistics

#### Sample Size Calculation

NRS scores were used as the main observational index in this study. In the pre-experiment stage, ultrasound-guided TPVB using the intercostal transverse and intercostal approaches were obtained. The mean of the NRS scores was 4.3 ± 1.2, 24 h after surgery. It was predicted that the ultrasound-guided parasagittal approach would be able to reduce the NRS scores by 1.3 points at 24 h postoperatively, reducing the pain degree to less than 3 points (NRS = 1–3 points, mild pain). The similar variance in two group is 5 and the effect size is 0.6. The α of the bilateral tests was 0.05 and the test power 1-β was 0.8. The Two-Sample Assuming Equal Variance Student's *t*-test was introduced *via* PASS15. The sample size of the two groups was 15 cases in each group. The sample size was increased as appropriate, and so a total of 34 patients were recruited.

SPSS24.0 was used for statistical analysis. All data were expressed by mean ± standard deviation (Mean ± SD). The Student's *t*-test was used to compare difference between two groups in measurement data, including age, weight, time, MAP, HR, and so on. In addition, the Mann-Whitney U-tests was used to assess statistical difference of block level and NRS between two groups. For the success ratio of catheterization between two groups, the Fisher exact probability method was performed to assess statistical difference because the sample size was <40. *P* < 0.05 was considered to be statistically significant.

## Results

A total of 34 patients, aged 45–77 years, were included in this study, but the observations were only completed for 23 of them, 14 of them being in group T, and 9 of them in group P (Figure 1). The sex, age, height, body mass, and weight of the two groups were compared, but the differences were not statistically significant (Table 1) (*P* > 0.05). The final success rate of the TPVB puncture was 100% in both groups. The TPVB catheterization time in group P was significantly longer than in group T (*P* < 0.05). After a single TPVB, the difference in block effect between the two groups was not found to be statistically significant (Table 1). The success rate of catheterization in group P was 64.7%, which was lower than in group T (94.1%), though there is no significant difference between two groups (*P* = 0.085, Table 2).

**Table 1:** General condition and partial observational indexes of both groups.

**Group**	**Group T** **(*n* = 17)**	**Group P** **(*n* = 17)**	***P*-value**
Gender (male/female)	10/7	8/9	0.732
Age (year)	60.9 ± 8.3	58.9 ± 8.5	0.521
Height (cm)	164.7 ± 7.9	163.3 ± 6.1	0.596
Weight (kg)	58.4 ± 10.1	58.5 ± 7.6	0.970
Operation time	217.3 ± 23.0	219.8 ± 22.9	0.802
BMI	21.4 ± 2.6	21.8 ± 2.6	0.682
Cathetering time (min)	10.9 ± 4.1	18.8 ± 6.3	0.001
Block effect (Excellent/Good/Poor)	13/4/0	12/5/0	1.000

*The cathetering time is calculated from the puncture and single block to cathetering*.

**Table 2:** Success ratio of cathetering comparison between the two groups.

	**Success (*n*)**	**Failure (*n*)**	**Success ratio (%)**	***P*-value**
Group T	16	1	94.1	0.085
Group P	11	6	64.7	

The differences between the two groups in MAP and HR were not statistically significant (*P* > 0.05) at the time of entering the operating theater, surgical skin incision, or 30 min after surgery (Table 3).

**Table 3:** MAP and HR comparison between the two groups at different time points.

**Group**	**Cases**	**Index**	**Entering in the** **operating room**	**Skin incision**	**30 min after** **cutting skin**
Group T	17	MAP (mmHg)	116.2 ± 9.4	108.4 ± 11.4	108.2 ± 12.2
		HR (bpm)	79.2 ± 10.7	78.5 ± 10.9	76.3 ± 9.8
Group P	17	MAP (mmHg)	118.3 ± 10.8	105.7 ± 14.6	104.5 ± 16.1
		HR (bpm)	78.6 ± 12.5	74.6 ± 10.1	76.3 ± 9.9

**Figure 2:**
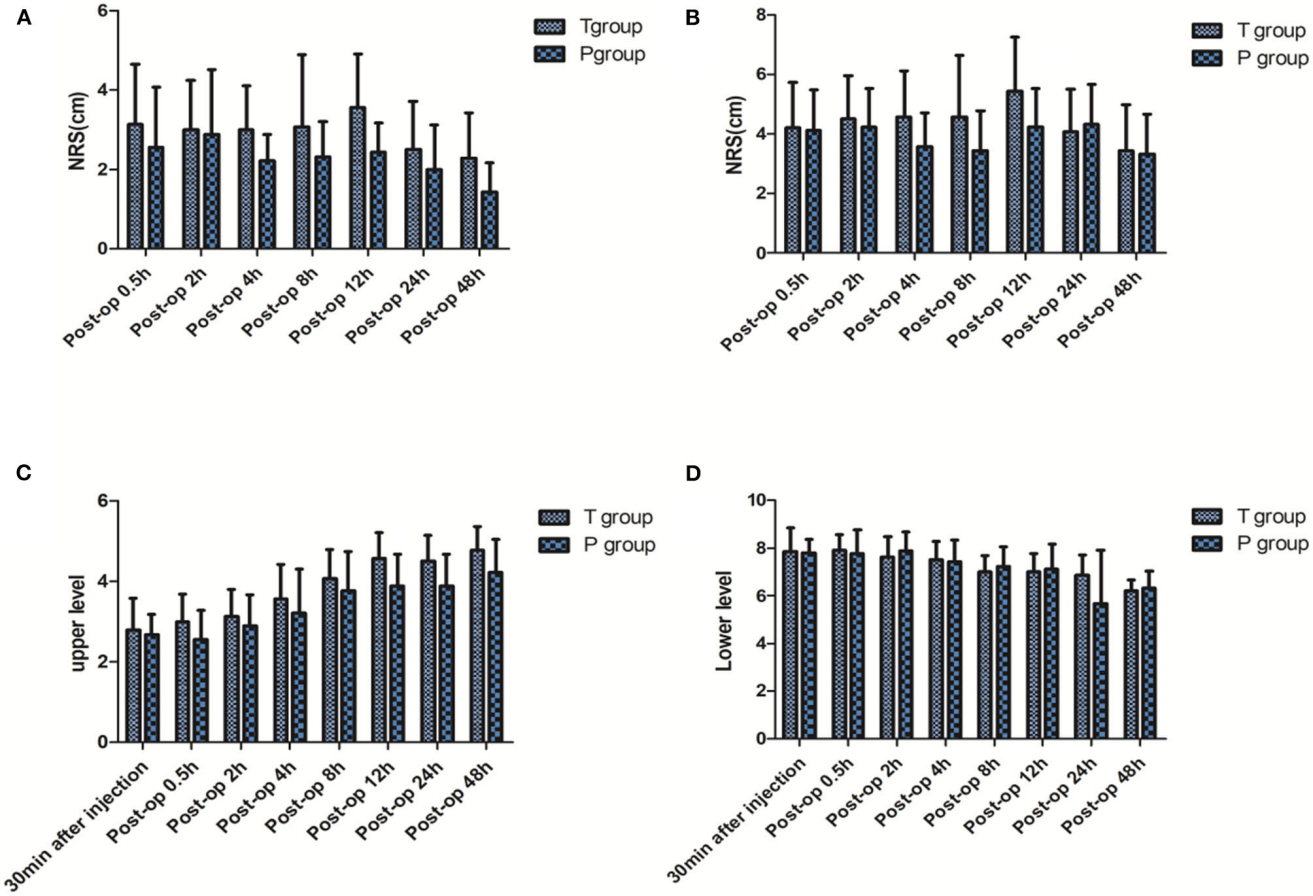
NRS at the postoperative time point of 0.5, 2, 4, 8, 12, 24, 48 h. **(A)** At rest; **(B)** during coughing. Block level at 30 min after injection and 0.5, 2, 4, 8, 12, 24, 48 h after surgery. **(C)** Upper level; **(D)** lower level. Post-op, Post operation. Compared with group T.

The difference in block effect between the two groups 30 min after giving TPVB at the surgical site was not statistically significant (*P* > 0.05). There were no signifificant differences in block level and NRS during coughing at the postoperative time point between the two groups (*P* > 0.05), but significant differences in NRS at rest (*P* < 0.05). Compared with the block level at 30 min after injection, the high block level was getting lower and the low block level was getting higher in the two groups at each time point after surgery (Figure 2). No case of pleural puncture, local anesthetic toxity, or intrathecal injection. There was no significant difference in the dosage of Flurbiprofen and Tramadols between the two groups (*P* > 0.05) (Table 4).

**Table 4:** The dosage of NSAIDs and Tramadol.

	**Group T** **(*n* = 14)**	**Group P** **(*n* = 9)**	***p-*value**
0~24 h Flurbiprofen (mg)	110.7 ± 21.3	111.1 ± 22.0	0.966
24~48 h Flurbiprofen (mg)	114.3 ± 30.6	122.2 ± 36.3	0.578
0–24 h Opioids (MME)	7.9 ± 8.6	4.7 ± 8.6	0.394
24–48 h Opioids (MME)	4.3 ± 4.5	2.4 ± 4.6	0.356

*Convert the dosage of Tramadol to MME. MME: milligrams of morphine equivalents*.

## Discussion

After thoracic surgery, the main causes of postoperative pain are the stimulation of the closed thoracic drainage tube and the pain after intercostal nerve injury. Although the thoracoscopic incision is small, the pain can still be so severe that the patient does not want to cough or is unable to do so. This can lead to pulmonary infection and atelectasis, preventing rapid recovery after surgery. The thoracic epidural block has always been the gold standard for postoperative analgesia in thoracic surgery. However, this technique is difficult to apply and prone to complications, such as hypotension, urinary retention, and total spinal anesthesia (3). As a result, some studies have proposed replacing it with TPVB because this has fewer complications. The paraverterble space refers to the wedge area of the lateral side of the intervertebral vertebra, which the lateral spinal nerve and sympathetic chain pass through. TPVB is the local anesthetic injection in this gap, which can not only block the spinal nerve, sympathetic chain, but even the local anesthetics can also spread to the adjacent paraverterble space and epidural cavity (4). Early TPVBs were mostly performed by single shot block with blind exploration method. Many studies have confirmed the effect of single shot block, but an indwelling catheter can be problematic and fail to provide continuous TPVB. Therefore, the effect of postoperative analgesia may be far less than that of a continuous epidural block. The ultrasound TPVB can not only display the position of puncture needle in the whole process, accurately display the paravertebral space, ensure the anesthetic effect, but also clearly display the position of pleura to avoid perforation of pleura, so it can improve the success rate of catheterization. In addition, some studies report that the paravertebral block using a thoracoscopic catheter-insertion technique is at least as effective as ultrasound-guided continuous TPVB for postoperative pain control. What's more, compared to ultrasound-guided catheter insertio, the thoracoscopic catheter-insertion technique will provide better safety and success rate because the paravertebral space filled with anesthetic during surgery will disturb the observation of ultrasound. Therefore, the thoracoscopic catheter-insertion technique maybe be a good anesthetic alternative (5, 6).

Prior to the study, we pre-tested the depth of the catheter placement. In the pre-test, two puncture approaches have been used for a single TPVB with a catheter inserted more than 6 cm and an opening made at 5 cm of the catheter. It was hoped that these methods would expand the diffusion of the liquid and increase the block levels. However, it has been found that catheterization is more difficult. International research has reported that if the catheter was placed into the space 2 cm beyond the needle tip, it is easier to insert into the intervertebral foramen, epidural space, or thoracic cavity (1, 7, 8). Fujii et al. (9) have also investigated the use of TPVB catheterization during a thoracoscopy. They found that it was difficult to push the catheter up over the rib in the puncture space, and as the depth of the catheter increased, it moved ahead less easily. As a result, this randomized controlled trial was set up to use a catheterization depth of 2 cm.

The dose of TPVB for the first time in the study was based on Miller anesthesiology 8th Edition (10), and the unpublished preliminary pre-test of sequential method was conducted. Through the pilot study, we obtain that the half effective volume of 0.3% ropivacaine in thoracoscopic surgery for lung cancer was 18.46 ml (95% CI 17.09~19.95 ml). Clinically, good results could be achieved by giving 1.1~1.2 times EV50, taking weight into account, finally we choose 0.33% ropivacaine 0.3 ml/kg as the first dose for single TPVB. There was no statistical difference between the two groups 30 min after the paravertebral injection, and all had a satisfactory block level. However, the failure rate of TPVB catheterization in group P was subsequently observed to be 35.3%, which was higher than the 5.9% failure rate in group T. Obviously, the success ratio in the group P is lower than that in the group T, although it was not statistically significant. The principal reason for no difference is lack of sample size and a larger sample of research should been used in further studies. The reasons for this are likely to have been as follows (11): the angle of inserting the needle was steep, and increased the difficulty of showing the needle by ultrasound. A double fulcrum effect was formed at the transverse process of the puncture segment and the ultrasonic probe of the parasagittal approach, which shortened the distance of the puncture needle from the ultrasonic probe and limited its adjustable angle. Moreover, the puncture needle had to pass through many groups of ligaments, and the pleural development was not as obvious as it was in group T. If the needle tip is blocked by even a small fiber, the catheterization will fail, and the adjustment of the puncture needle can easily break the pleura because of the limited distance, thereby leading to an increase in failure rates. All of these problems may lead to prolonged catheterization and explain the high failure rate of the parasagittal approach. A study on corpses by Paraskeuopoulos et al. (12) found that while the ultrasound-guided transversal intercostal approach within the plane required 1–4 attempts to puncture successfully, the parasagittal approach required 1–7 attempts.

This study has also found that there is no significant difference between the two groups in the block level and effect of the needle testing surgical site, skin incision, and MAP and HR 30 min after operation after using single TPVB, indicating that there is no significant difference in the effect of single TPVB block between the two groups. There was no significant difference in block level and NRS during coughing at each time point after successful catheterization between the two groups, and there was no significant difference in the dosage of NSAIDs and tramadol because of the NRS more than 4, suggesting that even sagittal and longitudinal catheterization could not improve the effect of continuous obstruction. The patients selected for this study underwent unilateral lung cancer surgery with a T_4_ and T_7_ operating hole. Therefore, the block plane had to reach T_4_-T_7_ to meet the needs of intraoperative and postoperative analgesia. Because the block plane did not meet the requirements of the surgery in the pre-test, the dosage of local anesthetic was gradually increased. As a result, the amount of ropivacaine used in this study reached the maximum dose of 800 mg/24 h (13). Furthermore, the parameter setting of the analgesic pump also reached its limit. Although the dosage of local anesthetic reached the maximum, the postoperative block level of the patients in the two groups became narrower with the passage of time, and the anesthetic segment no longer covered the operation field. The results of Helms et al. show that the continuous analgesia of paravertebral space is ineffective (14). The reasons why the postoperative continuous TPVB plane narrows may be that the continuous infusion of local anesthetics through analgesic pump may lead to the plane diffusion not being wide enough due to the slow infusion rate. The thoracic paravertebral space is an anatomically wedge-shaped space from the intervertebral foramen to the costal angle, and it is extensive and loose. Due to gravity, the liquid is mainly concentrated in the puncture segment. When the catheter position changes, a gap appears between the needle tip and the catheter tip (15). The narrowing of the block levels after continuous TPVB suggests that postoperative continuous TPVB may not meet analgesic requirements after thoracoscopic surgery.

Previous studies reported that paravertebral block had pneumothorax, pleural penetration, vascular puncture, and other complications (16), but these complications did not occur in the two groups in this study, which may be related to the application of ultrasound being used to avoid pleura, and the small sample size. Through the study, we found that a single TPVB performed under either the ultrasound-guided transverse intercostal approach or parasagittal approach can satisfy intraoperative analgesia. Moreover, there are no significant differences between the postoperative block level and pain scores during coughing when the two methods are used to provide continuous TPVB for postoperative analgesia after thoracoscopic surgery. In addition, catheterization with the parasagittal approach may take longer, and the failure rate is higher. However, the sample size of this study is small, and there were more dropouts from the test in group P because of catheterization failure, so it is not reasonable to draw firm conclusions, but this study at least provide a new reference between ultrasound-guided transverse intercostal approach and parasagittal approach.

## Data Availability Statement

The original contributions presented in the study are included in the article/supplementary material, further inquiries can be directed to the corresponding author.

## Ethics Statement

The studies involving human participants were reviewed and approved by Ethics Committee of Zhangzhou Affiliated Hospital of Fujian Medical University. The patients/participants provided their written informed consent to participate in this study.

## Author Contributions

Q-wH and Z-wL conceived the idea and conceptualized the study. Z-jL and Q-wH collected the data. J-bL and W-qZ analyzed the data. L-wJ and Z-jL drafted the manuscript. L-wJ and Z-wL reviewed the manuscript. All authors read and approved the final version of the manuscript.

## Conflict of Interest

The authors declare that the research was conducted in the absence of any commercial or financial relationships that could be construed as a potential conflict of interest.

## Publisher's Note

All claims expressed in this article are solely those of the authors and do not necessarily represent those of their affiliated organizations, or those of the publisher, the editors and the reviewers. Any product that may be evaluated in this article, or claim that may be made by its manufacturer, is not guaranteed or endorsed by the publisher.
